# Genome-Wide Association Study of Egg Production Traits in Shuanglian Chickens Using Whole Genome Sequencing

**DOI:** 10.3390/genes14122129

**Published:** 2023-11-25

**Authors:** Ming Fu, Yan Wu, Jie Shen, Ailuan Pan, Hao Zhang, Jing Sun, Zhenhua Liang, Tao Huang, Jinping Du, Jinsong Pi

**Affiliations:** 1 Institute of Animal Husbandry and Veterinary, Hubei Academy of Agricultural Science, Wuhan 430064, China; fuming19911203@163.com (M.F.); 15927657060@163.com (J.S.); panailuan08319@163.com (A.P.); 15172520011@163.com (H.Z.); sammi8866@sina.com (J.S.); liangzhenghua2046@163.com (Z.L.); huangtao214@126.com (T.H.); ddjinpin@163.com (J.D.); pijinsong@sina.com (J.P.); 2 Hubei Key Laboratory of Animal Embryo and Molecular Breeding, Hubei Academy of Agricultural Science, Wuhan 430064, China

**Keywords:** Shuanglian chickens, egg production trait, GWAS, whole genome sequencing

## Abstract

Egg production is the most important economic trait in laying hens. To identify molecular markers and candidate genes associated with egg production traits, such as age at first egg (AFE), weight at first egg (WFE), egg weight (EW), egg number (EN), and maximum consecutive egg laying days (MCD), a genome-wide analysis by whole genome sequencing was performed in Shuanglian chickens. Through whole genome sequencing and quality control, a total of 11,006,178 SNPs were obtained for further analysis. Heritability estimates ranged from moderate to high for EW (0.897) and MCD (0.632), and from low to moderate (0.193~0.379) for AFE, WFE, and EN. The GWAS results showed 11 genome-wide significant SNPs and 23 suggestive significant SNPs were identified to be associated with EN, MCD, WFE, and EW. Linkage disequilibrium analysis revealed twenty-seven SNPs associated with EN were located in a 0.57 Mb region on GGA10, and clustered into five blocks. Through functional annotation, three candidate genes *NEO1*, *ADPGK,* and *CYP11A1,* were identified to be associated with EN, while the *S1PR4*, *LDB2,* and *GRM8* genes was linked to MCD, WFE, and EW, respectively. These findings may help us to better understand the molecular mechanisms underlying egg production traits in chickens and contribute to genetic improvement of these traits.

## 1. Introduction

Egg production is the most important economic trait in laying hens. In 2022, global chicken egg production was approximately 88.68 million tons, accounting for about 93% of the poultry egg production [1]. Egg production traits mainly include the egg number (EN), age at first egg (AFE), weight at first egg (WFE), and egg weight (EW). EN is the most important production performance indicator among these traits, which is directly related to egg production [2,3]. Furthermore, improving these egg production traits is the main objective in chicken breeding programs [4]. However, since these traits are commonly of low heritability, complex, and controlled by polygenes, the genetic progress obtained through conventional breeding approaches is minor [5]. Therefore, it is necessary to identify the key markers and genes associated with these traits for molecular breeding.

QTL mapping and genome-wide association study (GWAS) have been widely employed to detect many molecular markers for complex traits in chickens [6,7,8]. To date, a total of 540 QTLs have been reported to be associated with egg number (EN), 56 QTLs with age at first egg (AFE), and 393 QTLs with egg weight (EW) in the Chicken QTL database (https://www.animalgenome.org/cgi-bin/QTLdb/GG/index, accessed on 25 August 2023). However, the resolution of QTL mapping and GWAS is poor due to the wide confidence intervals for position, resulting in few detected causative genes associated with these traits. In addition, studies on candidate genes related to egg production traits are mainly focused on the endocrine hypothalamic-pituitary-gonadal (HPG) axis [9]. A number of candidate genes, including *GnRH-I*, *NPY*, *GNRHR*, *GnIH*, *FSHR*, *PRL*, *PRLR*, *ESR,* and *VIP*, have been identified by these association studies [10]. In novel genes mining by GWAS, related studies have only reported a few SNPs significantly associated with egg production traits, and most of these SNPs are located on chromosomes 1, 2, 3, 4, 5, 6, 9, and 21 [9,11,12,13,14]. Therefore, to better understand the molecular mechanisms underlying egg production performance, more candidate genes and SNPs need to be identified.

The Shuanglian chicken is a well-known indigenous breed in China, which has characteristics of early sexual maturity, good meat quality, disease resistance, and excellent egg quality. In the present study, GWAS was performed on 163 Shuanglian chickens to identify molecular markers and candidate genes affecting egg production traits by whole genome sequencing. These promising SNPs and genes could be helpful to engineer practical breeding programs for the improvement of egg production performance.

## 2. Materials and Methods

### 2.1. Ethics Statement

All the animal research activities in this study were approved by the Animal Care and Use Committee of the Institute of Animal Science and Veterinary Medicine, Hubei Academy of Agricultural Sciences (HBAAS20220216).

### 2.2. Population and Phenotyping Measurement

The population size of Shuanglian chickens was 163 in this study. All chickens were raised in stair-step individual cages at Jinshui Experimental Base (Wuhan, Hubei province, China) under the regular feeding and management conditions. We recorded the egg production every day. The phenotyping measurement includes six traits: age at first egg (AFE), weight at first egg (WFE), egg weight (EW), egg number at 40 weeks (EN40), egg number at 43 weeks (EN43), and maximum consecutive egg laying days (MCD). The phenotypic correlation between each pair of traits were evaluated using SPSS software 20. A blood sample of each chicken was collected and put into a corning tube with EDTA for preparing the genomic DNA.

### 2.3. Whole Genome Sequencing and Quality Control

The genomic DNA was extracted using a standard technique of digestion with proteinase K followed by phenol-chloroform extraction and ethanol precipitation [15]. DNA concentration and purity were measured by NanoDrop 2000 Spectrophotometer (Thermo Scientific, Waltham, MA, USA). The DNA extract was stored at −80 °C for further analysis.

The genomic DNA of 163 chickens was sequenced in Beijing Compass Biotechnology Co. Ltd. (Beijing, China) High-quality paired-end 150 bp reads were obtained with a sequencing depth of 10× for each sample. All the reads were aligned against the GRCg7b reference (Ensembl release 110) using Burrow-Wheeler Alignment (BWA) software 0.1.17 [16]. The obtained reads were realigned using GATK software 4.1.8.1 [17]. The SNPs were then filtered with the following parameters: genotyping call rate < 0.95, minor allele frequency (MAF) < 0.05, and Hardy-Weinberg equilibrium (HWE) > 10^−6^ in PLINK1.90 [18]. The remaining SNPs with missing genotypes were imputed using the Beagle v5.0 procedure [19]. Finally, a total of 11,006,178 SNPs were obtained from 163 individuals.

### 2.4. Estimation of Genetic Parameters

Genetic parameters, including heritability and genetic correlation, were estimated based on average information-restricted maximum likelihood algorithm (AI-REML) in GCTA software 1.24.7 [20]. The estimate model was as follows:

y=Xb+Zu+e

where y is the vector of phenotype; b is the fixed effect; X and Z are the matrices corresponding to b and u; and e is the residual. u indicates the normal distribution ($u\sim N(0,\;G_{jk}\sigma_{u}^{2}))$ that all the SNPs obey, in which $\sigma_{u}^{2}$ is additive genetic variance, and $G_{jk}$ is constructed by genomic information:

$$G_{jk}=\frac{1}{N}\sum_{i=1}^{N}\frac{\left(X_{ij}-2p_{i})(X_{ik}-2p_{i}\right)}{2p_{i}(1-p_{i})}$$

where $X_{ij}$ is the copy number of the reference allele at the ith SNP of individual j; $X_{ik}$ is the copy number of the reference allele at the ith SNP of individual k; $p_{i}$ is the minor allele frequency of the i*^th^* SNP in the population; and N is the number of SNPs. The heritability of each trait was calculated as $h^{2}=\frac{\sigma_{g}^{2}}{\sigma_{g}^{2}+\sigma_{e}^{2}}$. Genetic correlation was calculated as:

$$r_{G}=\frac{\left({cov}_{G_{XY}}\right)}{\sqrt{\sigma_{G_{X}}^{2}\sigma_{G_{Y}}^{2}}}$$

where $r_{G}$ represents the genetic correlation between trait X and Y; ${cov}_{G_{XY}}$ indicates the covariance of two traits; and $\sigma_{G_{X}}^{2}$ and $\sigma_{G_{Y}}^{2}$ indicate the variances of two traits, respectively.

### 2.5. Genome-Wide Association Study

The genome-wide association analysis was performed by mixed linear model in the GEMMA software 0.98.3 [21], and the model was as follows.

y=Wa+Xb+u+e

where y is the vector of phenotype; W is a matrix of covariates (including a column of 1 s and top three principal components of population structure); a is a vector of the corresponding coefficients including the intercept; X is a vector of SNP genotypes; b is the effect size of the SNP; u is a vector of individual random effects; e is a vector of errors. *p*-values were adjusted by the Bonferroni multiple test method, the threshold *p*-values of suggestive and genome-wide significance were calculated as 9.09 × 10^−8^ (1/11,006,178) and 4.54 × 10^−9^ (0.05/11,006,178), respectively.

### 2.6. Variance Explained by the Most Significant SNPs

Based on the most significant SNP associated with each trait, a linear regression model was constructed for analysis of variance:

Y=a+bX+e

where Y is the phenotype; X is the genotype of the SNP; and b is the effect value of the genotype; a is the intercept which represents the background value of individual phenotype after deducting the genotype effect; and e is the residual. Therefore, there are three variances in this model, and their relationship was as follows:

$$Var\left(Y\right)=Var\left(bX\right)+Var\left(e\right)$$

where $Var\left(Y\right)$ is the variance of phenotype; $Var\left(bX\right)$ is the variance of genetic effect of the SNP; and $Var\left(e\right)$ is the variance of residual. The percentage of the phenotype variance explained by the most significant SNP was calculated as follows:

$$Var\left(SNP\right)\%=Var\left(bX\right)/Var\left(Y\right)$$

### 2.7. Linkage Disequilibrium Analysis and Identification of Candidate Genes

Linkage disequilibrium (LD) analysis was performed using LDBlockShow software 1.40 [22]. The candidate genes near the most significant SNPs or in the candidate genomic region were identified by the gene annotation information from Ensembl genome browser 110 (http://asia.ensembl.org/index.html, accessed on 25 August 2023). Kyoto Encyclopedia of Genes and Genomes (KEGG) and Gene Ontology (GO) analyses were employed to identify related pathways. KEGG and GO analyses were performed using OmicShare Tools (https://www.omicshare.com, accessed on 25 August 2023).

## 3. Results

### 3.1. Phenotype Data, Whole Genome Sequencing Data, and Population Structure

**Figure 1:**
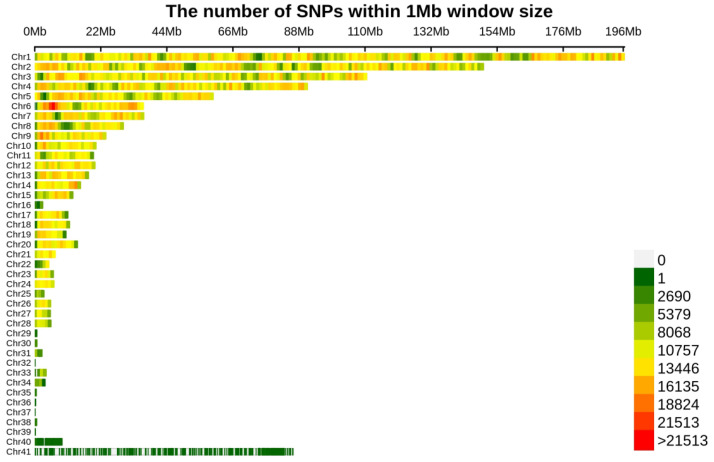
The density plot of SNPs.

The phenotype data of age at first egg (AFE), weight at first egg (WFE), egg weight (EW), egg number at 40 weeks (EN40), egg number at 43 weeks (EN43), and maximum consecutive egg laying days (MCD) in the Shuanglian chicken population are shown in Table 1. Except for EW, the coefficient of variation (CV) of all traits is above 10%, with MCD having the highest CV (54.3%). Through whole genome sequencing and quality control, a total of 11,006,178 high-quality SNPs were obtained. The SNP density plot is presented in Figure 1. Principal component analysis (PCA) was carried out. And, the scatter plot of the first two principal components is displayed in Figure S1.

### 3.2. Estimation of Phenotypic and Genetic Parameters

Heritability, genetic correlation, and phenotypic correlations for AFE, WFE, EW, EN40, EN43, and MCD are shown in Table 2. Heritability of AFE, WFE, EW, EN40, EN43, and MCD was estimated to be within 0.193 and 0.897, with the heritability of egg weight (0.897) being the highest among them. The phenotypic correlation data indicated there was a significant correlation between most traits, AFE had a highly significant positive correlation with EW, and a highly significant negative correlation with EN. Similarly, a strong genetic correlation was observed among EN40, EN43, and MCD, the genetic correlation coefficient was almost 1, while the phenotypic correlation coefficient was also above 0.694.

The phenotypic correlation coefficients and genetic correlation coefficients among traits were located in the upper and lower triangle, respectively; the heritability of each trait was located in diagonal of the table. * indicates significant correlation (*p* < 0.05), ** (*p* < 0.01) and *** (*p* < 0.001) indicates extremely significant correlation, respectively.

### 3.3. Genome-Wide Association Analysis

**Figure 2:**
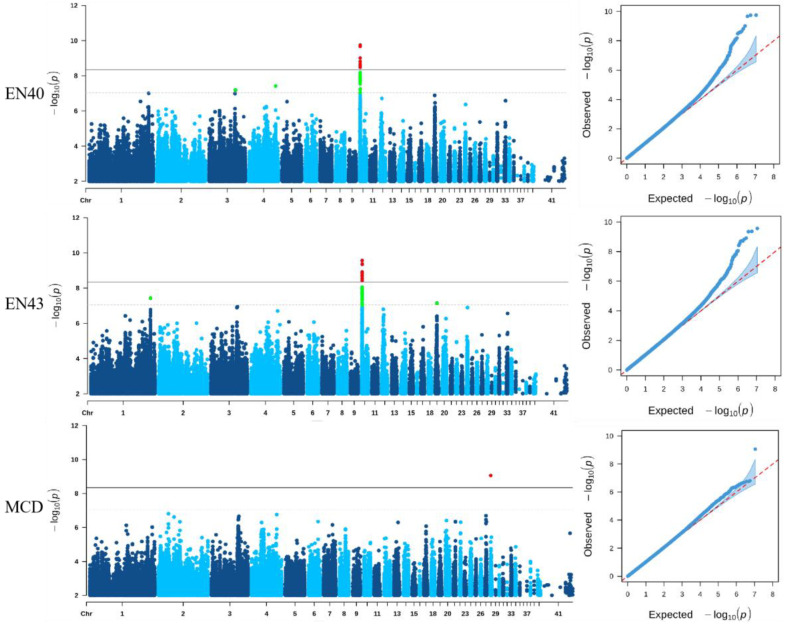
Manhattan plots and quantile-quantile (Q-Q) plots of egg number at 40 weeks (EN40), egg number at 43 weeks (EN43), and maximum consecutive egg laying days (MCD). The solid and dashed line indicates the genome-wide and suggestive significance threshold, respectively. Red points indicate the genome-wide significant SNPs, green points indicate the suggestive significant SNPs.

The GWAS results of EN40, EN43, and MCD are shown in Figure 2, and the information of the significant SNPs is illustrated in Table 3.

GWAS results indicated 10 SNPs significantly associated with EN40 and EN43 were detected on chromosome 10 (GGA10), and the significant SNPs detected for these two traits were completely consistent, covering a 0.19 Mb (1.77 Mb−1.96 Mb) genomic region. The most significant SNP of EN40 was rs794599852 (G > A), located in the intron of *NEO1* gene, and it explained 8.5% of the phenotypic variance. The most significant SNP of EN43 were rs737101872 (A > G), located in the intron of the *ENSGALG00010025119* gene, and it explained 7.2% of the phenotypic variance. The most significant SNP of MCD was 28:1696604 (A > G), located in the upstream of the *S1PR4* gene on GGA28, and it explained 22.8% of the phenotypic variance. In addition, nineteen suggestive significant SNPs were also found on GGA3, GGA4, and GGA10 to be associated with EN40; seventeen suggestive significant SNPs were also found on GGA1, GGA10, and GGA19 to be associated with EN43; one suggestive significant SNP related to EW and WFE was found on GGA1 and GGA4 (Figure S2, Table 4), respectively. Unfortunately, there was no genome-wide and suggestive significant SNP associated with AFE.

### 3.4. Linkage Disequilibrium Analysis

**Figure 3:**
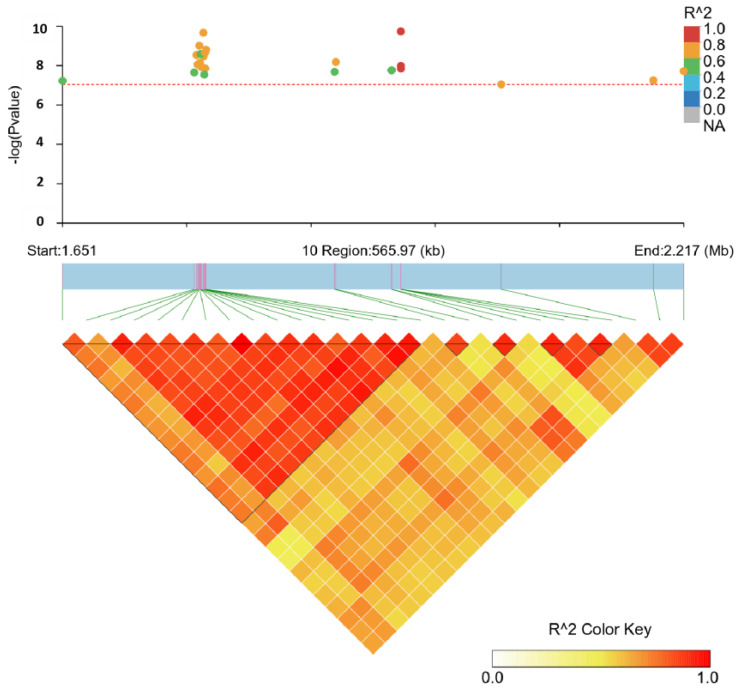
Linkage disequilibrium analysis of genomic regions associated with EN.

In order to fine-map the genomic region associated with EN, linkage disequilibrium analysis of the associated SNPs (10 significantly and 17 suggestively) on GGA10 was performed using LDBlockShow software 1.40 in Figure 3. The LD analysis revealed all genome-wide and suggestive significant SNPs associated with EN were located in the 0.57 Mb region, which were in strong LD status and were clustered into five blocks. Gene annotation data showed ten candidate genes (*CELF6*, *BBS4*, *ADPGK*, *HEXA*, *ARIH1*, *PARP6*, *NEO1*, *HCN4*, *REC114,* and *NPTN*) were identified in the block region.

### 3.5. Enrichment Analysis

**Figure 4:**
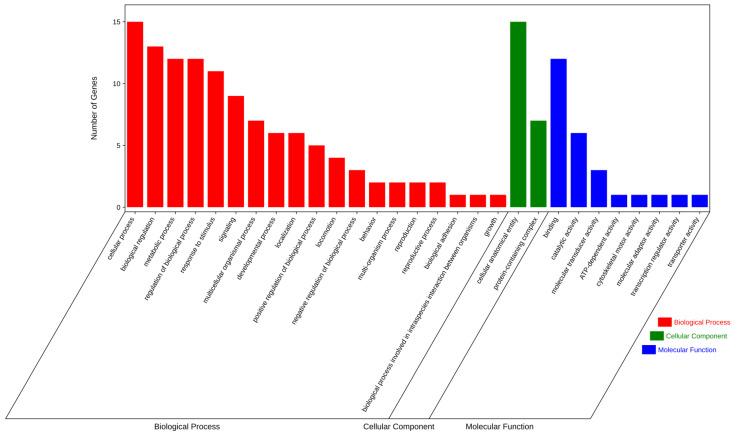
GO annotation analysis for candidate genes.

**Figure 5:**
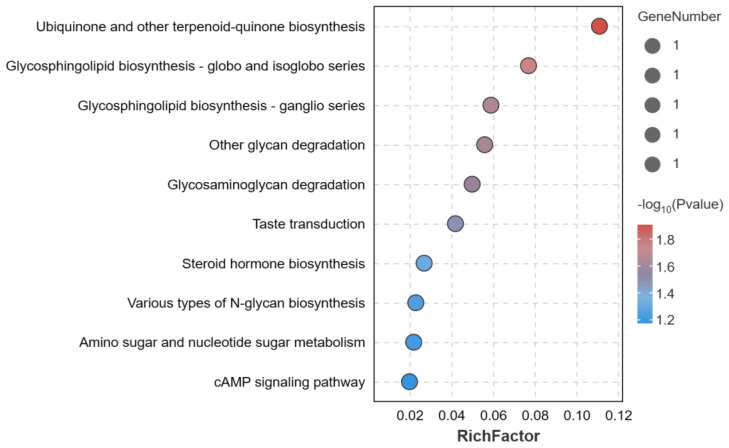
KEGG pathway analysis for candidate genes.

To gain insight into the function of these 34 significant SNPs associated with egg production traits, the corresponding candidate genes were used for GO and KEGG enrichment analysis. Most of these genes were significantly enriched in GO terms of biological processes (BP) and participated in cellular process (Figure 4). KEGG analysis revealed a total of seven significantly enriched pathways, mainly including the ubiquinone and other terpenoid-quinone biosynthesis, glycosphingolipid biosynthesis, and steroid hormone biosynthesis (Figure 5). More details of the GO and KEGG enrichment can be found in Tables S1 and S2.

## 4. Discussion

Egg production is not only an important economic trait, but also a complex quantitative trait, which is influenced by both genetics and environment, with genetics being the determining factor [9]. In this study, we present a GWAS to identify the candidate genes associated with egg production traits in a Shuanglian chicken population from China. In general, the important conditions for GWAS to achieve better results mainly include the number of markers, population structure, and relationships between individuals [23]. With the continuous development of high-throughput sequencing technology, the application of whole genome resequencing is becoming increasingly widespread. In this study, whole genome sequencing was used to obtain more genetic variation information. A total of 11,006,178 high-quality SNPs were finally obtained through quality control, which were evenly distributed on each chromosome. Based on high-quality SNPs, we analyzed the population structure and the relationship between individuals. The analysis of population structure indicated the population roughly fell into three subgroups. Therefore, in the construction of the GWAS model, the first three principal components from PCA were used as covariates, and the genetic relationship between individuals was also taken into consideration.

The coefficient of variation is an important indicator for measuring the uniformity of traits. In this study, the CV of AFE, WFE, MCD, EN40, and EN43 were all above 10%; the CV of MCD was 54.3%, indicating the significant phenotypic variations in these traits. In general, heritability is considered low if the value varies from 0 to 0.20, moderate at 0.20 to 0.40, and high at >0.40. Previous studies have shown the heritability of egg production traits was mostly between 0.160 and 0.640 [24,25], with the heritability of EN ranging from 0.160 to 0.250, indicating a low to moderate level of heritability. In this study, apart from EW (0.897), the heritability of AFE, WFE, MCD, EN40, and EN43 was within 0.193 and 0.632, indicating our estimation results of trait heritability were comparable to previous research. The high heritability of EW in this study indicated the genetic factor accounted for a large proportion of the phenotypic variation, which was conducive to revealing genetic characteristics of this trait in Shuanglian chickens.

The genetic correlation coefficient between AFE and EW, EN40, EN43, MCD were 0.678, −0.764, −0.789, and −0.442, respectively, suggesting a strong correlation among these traits. Moreover, the results of phenotypic correlation were consistent with those of genetic correlation, and there was a significant correlation between these traits, which indicated AFE could be used as a key indicator for predicting the EN. MCD can reflect the continuous production performance of the chicken population [26]; the genetic correlation coefficient between EN and MCD was almost 1 in this study, suggesting the higher the number of eggs produced by the population, the better the continuous production performance. Therefore, the AFE and MCD could be considered important indicators in the subsequent breeding of Shuanglian chickens, while balancing the WFE for joint breeding to further improve its production performance.

GWAS results indicated all the significant SNPs associated with EN40 and EN43 were located on chromosome 10, and these SNPs were all located in regulatory regions such as intron and intergenic regions, indicating regulatory mutation might be the determinants of phenotypic differences. In addition, the proportion of the phenotypic variance explained by the most significant SNP of EN40, EN43, and MCD was 8.5%, 7.2%, and 22.8%, respectively, implying our method for the identification of main candidate genes associated with these traits was powerful and effective. After LD analysis, all the genome-wide and suggestive significant SNPs associated with EN located in the 0.57 Mb region, which were in strong LD status and were clustered into five blocks. These blocks contained a total of 10 candidate genes: *CELF6*, *BBS4*, *ADPGK*, *HEXA*, *ARIH1*, *PARP6*, *NEO1*, *HCN4*, *REC114,* and *NPTN.* Through functional annotation, two candidate genes *NEO1* and *ADPGK* were reported to be associated with reproductive performance. It was found the *NEO1* gene was closely related to the fertility of humans and mice, and this gene was highly expressed in both ovaries and uterus [27,28]. The amino acid homology encoded by this gene in mice and chickens is as high as 94%, and the expression pattern of chicken *NEO1* is similar to that of mouse [29]. *ADPGK* plays an important role instead of hexokinase in regulating energy metabolism [30]. It has also been confirmed to be related to the reproductive performance [31]. In addition, we also annotated the upstream and downstream 1 Mb regions of all genome-wide and suggestive significant SNPs and identified an important candidate gene *CYP11A1* related to egg production performance. The *CYP11A1* gene is the first hydrolytic enzyme that catalyzes the production of steroid hormones and is also the rate-limiting enzyme of this process. It plays an important role in the reproductive physiology of animals regulated by hormones [32]. According to previous studies, the *LDB2* gene could regulate cell migration, and a 31-bp Indel within *LDB2* gene was significantly associated with growth traits in the F2 resource chicken population and affected the expression of *LDB2* in muscle tissue [33,34,35]. Furthermore, in studies with Lingxian white goose and goats, the *LDB2* gene was also confirmed to be associated with growth and weight traits [36,37,38]. The above studies indicate the *LDB2* gene is closely related to the weight traits of poultry and can be an important candidate gene for WFE of Shuanglian chickens in our study. For the EW trait, the *GRM8* gene encodes a G protein-coupled receptor, involved in glutamatergic neurotransmission in the central nervous system, and it plays a role in intracellular signal transduction and cyclic adenylate signal cascade inhibition [39]. Previous studies found the *GRM8* gene could participate in the generation of *GABA*, an inhibitory neurotransmitter in the brain and associated with egg weight [40,41]. In addition, the *S1PR4* gene is a signaling lipid. It could regulate cell fate and participate in STAT3, MAPK, and Akt pathways [42], which were involved in regulating the cell cycle, proliferation and apoptosis of chicken follicular granulosa cells [43]. Perhaps, the *S1PR4* and *GRM8* genes may be potential candidate genes for MCD and EW traits, respectively.

## 5. Conclusions

In conclusion, the GWAS performed by whole genome sequencing in this study demonstrated 34 significant SNPs were identified to be associated with EN, MCD, WFE, and EW. Moreover, three genes *NEO1*, *ADPGK*, and *CYP11A1* were identified as candidates associated with EN, while the *S1PR4*, *LDB2*, and *GRM8* genes were linked to MCD, WFE, and EW, respectively. Our findings may help us to better understand the molecular mechanisms underlying egg production traits in chickens and contribute to genetic improvement of these traits.

## Figures and Tables

**Table 1:** The phenotype data of AFE, WFE, EW, EN40, EN43, and MCD.

Traits	N	Mean ± SD	Minimum	Maximum	CV
AFE (d)	163	131.7 ± 14.8	106	169	11.2
WFE (g)	163	1491.8 ± 195.1	1060	2020	13.1
EW (g)	163	48.4 ± 4.0	38.0	64.6	8.2
EN40	163	111.3 ± 25.5	29	182	22.9
EN43	163	125.6 ± 28.0	29	165	22.3
MCD (d)	163	14.6 ± 7.9	3	49	54.3

SD, standard deviation; CV, coefficient of variation (%).

**Table 2:** Estimation of phenotypic and genetic parameters with AFE, WFE, EW, EN40, EN43, and MCD.

Traits	AFE	WFE	EW	EN43	EN40	MCD
AFE	0.193	−0.006	0.283 ***	−0.474 ***	−0.512 ***	−0.234 **
WFE	−0.01	0.379	0.208 **	−0.311 ***	−0.304 ***	−0.269 ***
EW	0.678	0.338	0.897	−0.257 ***	−0.261 ***	−0.164 *
EN43	−0.764	−0.695	−0.283	0.246	0.991 ***	0.702 ***
EN40	−0.789	−0.704	−0.277	1	0.242	0.694 ***
MCD	−0.442	−0.429	−0.15	1	1	0.632

**Table 3:** Information of the genome-wide significant SNPs associated with EN40, EN43, and MCD.

Trait	Chr	SNP	Pos (bp) ^1^	MAF ^2^	Consequence	*p*-Value	Candidate Gene
EN40	10	rs794599852	1,959,133	0.135	intron variant	1.79 × 10^−10^	*NEO1*
	10	rs794410298	1,959,103	0.138	intron variant	1.84 × 10^−10^	*NEO1*
	10	rs737101872	1,779,403	0.153	intron variant	2.16 × 10^−10^	*ENSGALG00010025119*
	10	rs733757787	1,775,498	0.150	intron variant	9.77 × 10^−10^	*ENSGALG00010025119*
	10	rs733219775	1,781,689	0.147	intron variant	1.54 × 10^−9^	*ENSGALG00010025119*
	10	rs732611081	1,780,940	0.150	intron variant	2.19 × 10^−9^	*ENSGALG00010025119*
	10	rs737141396	1,776,849	0.141	intron variant	2.63 × 10^−9^	*ENSGALG00010025119*
	10	rs732388748	1,776,854	0.141	intron variant	2.63 × 10^−9^	*ENSGALG00010025119*
	10	rs738796503	1,773,085	0.156	intron variant	2.91 × 10^−9^	*ENSGALG00010025119*
	10	rs732783139	1,779,757	0.137	intron variant	3.31 × 10^−9^	*ENSGALG00010025119*
EN43	10	rs737101872	1,779,403	0.153	intron variant	2.72 × 10^−10^	*ENSGALG00010025119*
	10	rs794410298	1,959,103	0.138	intron variant	4.30 × 10^−10^	*NEO1*
	10	rs794599852	1,959,133	0.135	intron variant	4.52 × 10^−10^	*NEO1*
	10	rs733757787	1,775,498	0.15	intron variant	1.21 × 10^−9^	*ENSGALG00010025119*
	10	rs733219775	1,781,689	0.147	intron variant	1.45 × 10^−9^	*ENSGALG00010025119*
	10	rs737141396	1,776,849	0.141	intron variant	1.93 × 10^−9^	*ENSGALG00010025119*
	10	rs732388748	1,776,854	0.141	intron variant	1.93 × 10^−9^	*ENSGALG00010025119*
	10	rs732611081	1,780,940	0.15	intron variant	2.03 × 10^−9^	*ENSGALG00010025119*
	10	rs738796503	1,773,085	0.156	intron variant	2.86 × 10^−9^	*ENSGALG00010025119*
	10	rs732783139	1,779,757	0.137	intron variant	3.78 × 10^−9^	*ENSGALG00010025119*
MCD	28	28:1696604	1,696,604	0.064	upstream_gene_variant	8.70 × 10^−10^	*S1PR4*

^1^ Pos (bp) indicates SNP position derived from the GRCg7b reference (Ensembl release 110). ^2^ MAF indicates the minor allele frequency of first listed marker.

**Table 4:** Information of the suggestive significant SNPs associated with EN, WFE, and EW.

Trait	Chr	SNP	Pos (bp) ^1^	MAF ^2^	Consequence	*p*-Value	Candidate Gene
EN40	3	rs733670986	80,457,964	0.064	intron variant	6.45 × 10^−8^	*FILIP1*
	4	rs14498714	82,228,007	0.095	intron variant	3.81 × 10^−8^	*ZFYVE28*
	10	10:1899911	1,899,911	0.12	intron variant	6.60 × 10^−9^	*NEO1*
	10	rs732015286	1,776,094	0.147	intron variant	7.15 × 10^−9^	*ENSGALG00010025119*
	10	rs737315097	1,775,174	0.16	intron variant	8.72 × 10^−9^	*ENSGALG00010025119*
	10	rs14000416	1,774,142	0.16	splice donor variant	8.77 × 10^−9^	*ENSGALG00010025119*
	10	rs794011707	1,959,182	0.135	intron variant	1.04 × 10^−8^	*NEO1*
	10	rs735135718	1,777,599	0.154	intron variant	1.19 × 10^−8^	*ENSGALG00010025119*
	10	rs740745767	1,781,067	0.141	intron variant	1.39 × 10^−8^	*ENSGALG00010025119*
	10	rs794188332	1,959,213	0.142	intron variant	1.42 × 10^−8^	*NEO1*
	10	rs737509451	1,950,938	0.163	intron variant	1.69 × 10^−8^	*NEO1*
	10	rs737799627	1,950,648	0.175	intron variant	1.73 × 10^−8^	*NEO1*
	10	rs740728260	2,216,957	0.104	intron variant	1.89 × 10^−8^	*NPTN*
	10	10:1898764	1,898,764	0.104	intron variant	2.06 × 10^−8^	*NEO1*
	10	rs732281842	1,770,938	0.123	noncoding transcript exon variant	2.22 × 10^−8^	*ENSGALG00010025119*
	10	rs735734105	1,780,269	0.141	intron variant	2.89 × 10^−8^	*ENSGALG00010025119*
	10	10:2189290	2,189,290	0.104	intergenic variant	5.62 × 10^−8^	*HCN4 REC114*
	10	10:1650989	1,650,989	0.126	intergenic variant	5.97 × 10^−8^	*CELF6 HEXA*
	10	rs732724738	2,050,616	0.12	upstream gene variant	8.93 × 10^−8^	*HCN4*
EN43	1	rs733976198	182,156,165	0.068	intron variant	3.71 × 10^−8^	*DYNC2H1*
	10	rs737315097	1,775,174	0.16	intron variant	8.86 × 10^−9^	*ENSGALG00010025119*
	10	rs14000416	1,774,142	0.16	splice donor variant	9.13 × 10^−9^	*ENSGALG00010025119*
	10	rs732015286	1,776,094	0.147	intron variant	1.11 × 10^−8^	*ENSGALG00010025119*
	10	rs794011707	1,959,182	0.135	intron variant	1.52 × 10^−8^	*NEO1*
	10	rs735135718	1,777,599	0.154	intron variant	1.54 × 10^−8^	*ENSGALG00010025119*
	10	10:1899911	1,899,911	0.12	intron variant	1.63 × 10^−8^	*NEO1*
	10	rs740745767	1,781,067	0.141	intron variant	2.02 × 10^−8^	*ENSGALG00010025119*
	10	rs735734105	1,780,269	0.141	intron variant	2.15 × 10^−8^	*ENSGALG00010025119*
	10	rs794188332	1,959,213	0.142	intron variant	2.22 × 10^−8^	*NEO1*
	10	rs737509451	1,950,938	0.163	intron variant	2.34 × 10^−8^	*NEO1*
	10	10:1898764	1,898,764	0.104	intron variant	2.76 × 10^−8^	*NEO1*
	10	rs737799627	1,950,648	0.175	intron variant	3.54 × 10^−8^	*NEO1*
	10	rs732281842	1,770,938	0.123	noncoding transcript exon variant	4.61 × 10^−8^	*ENSGALG00010025119*
	10	10:1650989	1,650,989	0.126	intergenic variant	6.88 × 10^−8^	*CELF6 HEXA*
	10	rs740728260	2,216,957	0.104	intron variant	9.07 × 10^−8^	*NPTN*
	19	rs15845543	5,400,291	0.27	intron variant	7.23 × 10^−8^	*VKORC1L1*
WFE	4	rs317659920	75,866,861	0.389	intron variant	1.62 × 10^−8^	*LDB2*
EW	1	rs736022615	21,149,255	0.104	intron variant	5.55 × 10^−8^	*GRM8*

^1^ Pos (bp) indicates SNP position derived from the GRCg7b reference (Ensembl release 110). ^2^ MAF indicates the minor allele frequency of first listed marker.

## Data Availability

Data are contained within the article and supplementary materials.

## Supplementary Materials

The following supporting information can be downloaded at: https://www.mdpi.com/article/10.3390/genes14122129/s1. Figure S1. The analysis of principal component. Figure S2. The GWAS result of AFE, WFE, and EW. Table S1. Enrichment analysis of candidate genes for egg production traits by GO. Table S2. Enrichment analysis of candidate genes for egg production traits by KEGG.
