# Supplementary material for: Genome-Wide Association Study of Egg Production Traits in Shuanglian Chickens Using Whole Genome Sequencing
Source: Genes (Basel). 2023 Nov 25;14(12):2129. doi: 10.3390/genes14122129 (PMC10742582; doi:10.3390/genes14122129)
Supplement: Supplementary file 1 [file genes-14-02129-s001.zip › Supplementary files/Table S1.pdf]

Table S1. Enrichment analysis of candidate genes for egg production traits by GO.

GO Term	GO ID	Description	Number of genes	Genes
Biological Process	GO:0051703	biological process involved in intraspecies interaction between organisms	1	*BBS4*
Biological Process	GO:0050896	response to stimulus	11	*GRM8,DYNC2H1,ZFYVE28,CYP11A1,HEXA,BBS4,NEO1,HCN4,NPTN,S1PR4,VKORC1L1*
Biological Process	GO:0023052	signaling	9	*GRM8,DYNC2H1,ZFYVE28,HEXA,BBS4,NEO1,HCN4,NPTN,S1PR4*
Biological Process	GO:0040011	locomotion	4	*LDB2,BBS4,NEO1,NPTN*
Biological Process	GO:0007610	behavior	2	*HEXA,BBS4*
Biological Process	GO:0065007	biological regulation	13	*GRM8,DYNC2H1,LDB2,ZFYVE28,CYP11A1,CELF6,HEXA,ARIH1,BBS4,NEO1,HCN4,NPTN,S1PR4*
Biological Process	GO:0051704	multi-organism process	2	*HEXA,BBS4*
Biological Process	GO:0050789	regulation of biological process	12	*GRM8,DYNC2H1,LDB2,ZFYVE28,CELF6,HEXA,ARIH1,BBS4,NEO1,HCN4,NPTN,S1PR4*
Biological Process	GO:0008152	metabolic process	12	*DYNC2H1,LDB2,ZFYVE28,CYP11A1,PARP6,CELF6,HEXA,ARIH1,BBS4,ADPGK,NPTN,VKORC1L1*

Biological Process	GO:0022414	reproductive process	2	*HEXA,BBS4*
Biological Process	GO:0000003	reproduction	2	*HEXA,BBS4*
Biological Process	GO:0032501	multicellular organismal process	7	*DYNC2H1,LDB2,HEXA,BBS4,NEO1,HCN4,NPTN*
Biological Process	GO:0032502	developmental process	6	*DYNC2H1,LDB2,HEXA,BBS4,NEO1,NPTN*
Biological Process	GO:0051179	localization	6	*DYNC2H1,LDB2,HEXA,BBS4,HCN4,NPTN*
Biological Process	GO:0040007	growth	1	*BBS4*
Biological Process	GO:0048518	positive regulation of biological process	5	*DYNC2H1,LDB2,ARIH1,BBS4,NEO1*
Biological Process	GO:0022610	biological adhesion	1	*NPTN*
Biological Process	GO:0009987	cellular process	15	*GRM8,DYNC2H1,LDB2,ZFYVE28,CYP11A1,PARP6,CELF6,HEXA,ARIH1,BBS4,NEO1,HCN4,NPTN,S1PR4,VKORC1L1*
Biological Process	GO:0048519	negative regulation of biological process	3	*ZFYVE28,BBS4,NPTN*
Molecular Function	GO:0003774	cytoskeletal motor activity	1	*DYNC2H1*
Molecular Function	GO:0060089	molecular transducer activity	3	*GRM8,NEO1,S1PR4*
Molecular Function	GO:0060090	molecular adaptor activity	1	*BBS4*
Molecular Function	GO:0140657	ATP-dependent activity	1	*DYNC2H1*
Molecular Function	GO:0003824	catalytic activity	6	*CYP11A1,PARP6,HEXA,ARIH1,ADPGK,VKORC1L1*

Molecular Function	GO:0005488	binding	12	*DYNC2H1,LDB2,ZFYVE28,CYP11A1,CELF6,HEXA, ARIH1,BBS4,NEO1,HCN4,NPTN,VKORC1L1*
Molecular Function	GO:0005215	transporter activity	1	*HCN4*
Molecular Function	GO:0140110	transcription regulator activity	1	*LDB2*
Cellular Component	GO:0032991	protein-containing complex	7	*DYNC2H1,LDB2,HEXA,ARIH1,BBS4,NEO1,HCN4*
Cellular Component	GO:0110165	cellular anatomical entity	15	*GRM8,FILIP1,DYNC2H1,LDB2,ZFYVE28,CYP11A1, HEXA,ARIH1,BBS4,ADPGK,NEO1,HCN4,NPTN,S1 PR4,VKORC1L1*
