# Supplementary material for: Genome-Wide Association Study of Egg Production Traits in Shuanglian Chickens Using Whole Genome Sequencing
Source: Genes (Basel). 2023 Nov 25;14(12):2129. doi: 10.3390/genes14122129 (PMC10742582; doi:10.3390/genes14122129)
Supplement: Supplementary file 1 [file genes-14-02129-s001.zip › Supplementary files/Table S2.pdf]

Table S2. Enrichment analysis of candidate genes for egg production traits by KEGG.

KEGG Pathway	Pathway ID	Gene number	Pvalue	Gene
Ubiquinone and other terpenoid-quinone biosynthesis	ko00130	1	0.01217298	*VKORC1L1*
Glycosphingolipid biosynthesis - globo and isoglobo series	ko00603	1	0.01754078	*HEXA*
Glycosphingolipid biosynthesis - ganglio series	ko00604	1	0.02288264	*HEXA*
Other glycan degradation	ko00511	1	0.02421407	*HEXA*
Glycosaminoglycan degradation	ko00531	1	0.02687208	*HEXA*
Taste transduction	ko04742	1	0.0321688	*HCN4*
Steroid hormone biosynthesis	ko00140	1	0.04920662	*CYP11A1*
Various types of N-glycan biosynthesis	ko00513	1	0.05697991	*HEXA*
Amino sugar and nucleotide sugar metabolism	ko00520	1	0.06084533	*HEXA*
cAMP signaling pathway	ko04024	1	0.06597731	*HCN4*
