# Supplementary figures and images for: Genome-Wide Association Study of Egg Production Traits in Shuanglian Chickens Using Whole Genome Sequencing
Source: Genes (Basel). 2023 Nov 25;14(12):2129. doi: 10.3390/genes14122129 (PMC10742582; doi:10.3390/genes14122129)

# PCA

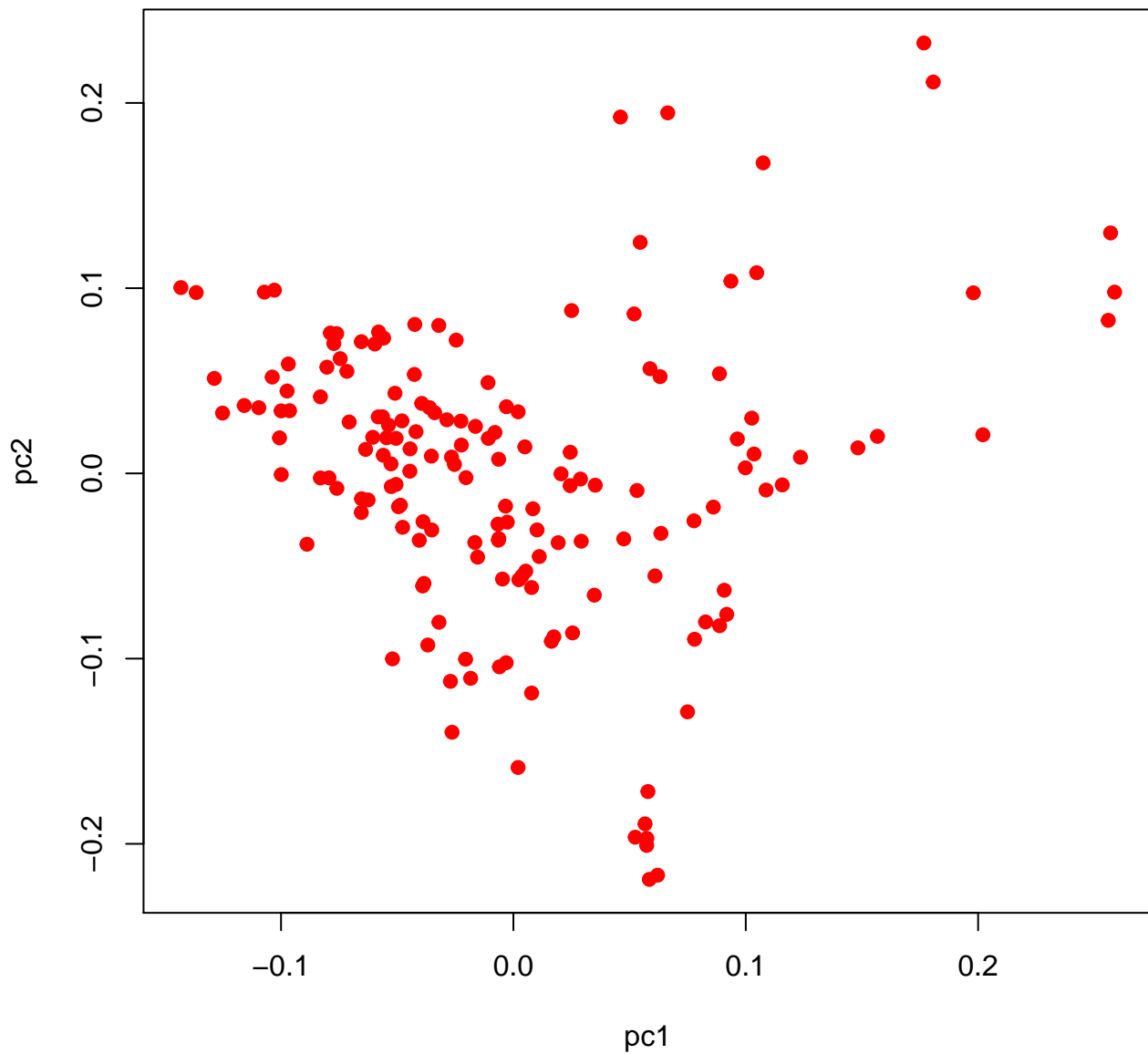

Supplement: Supplementary file 1 [file genes-14-02129-s001.zip › Supplementary files/Figure S1.pdf]

AFE

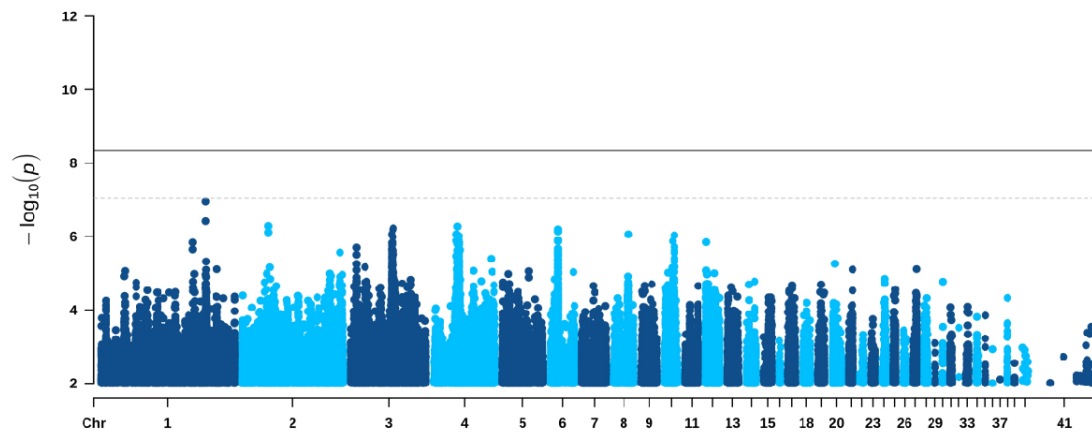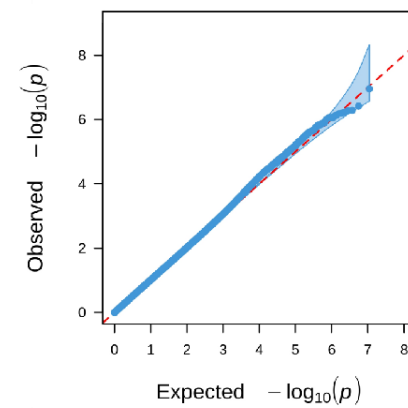

WFE

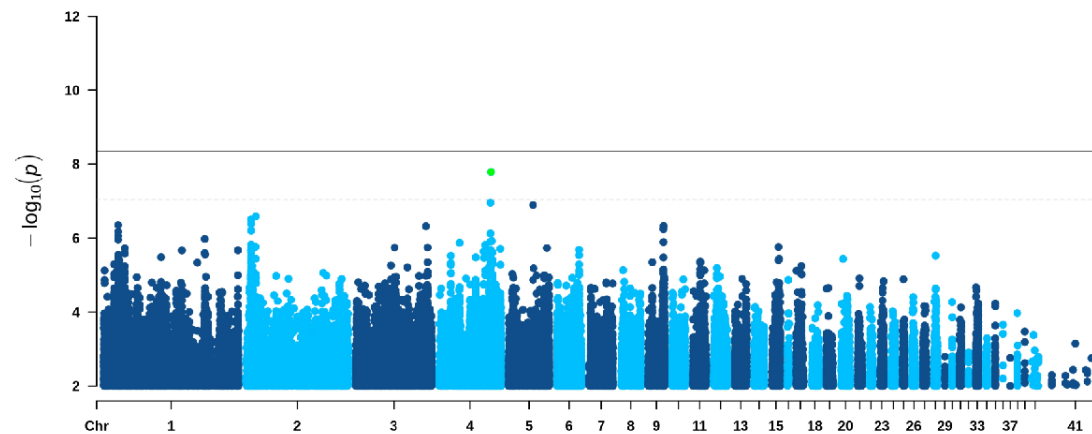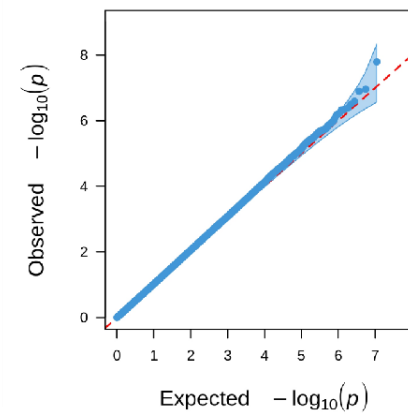

EW

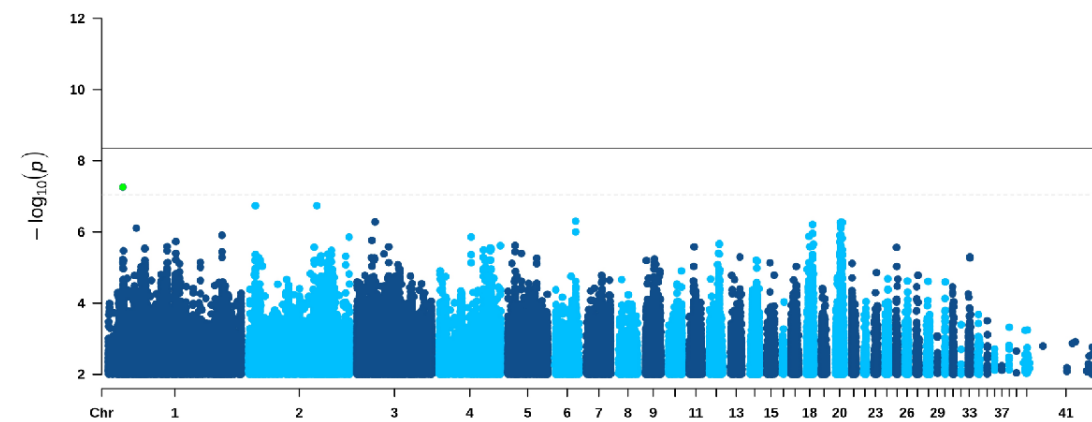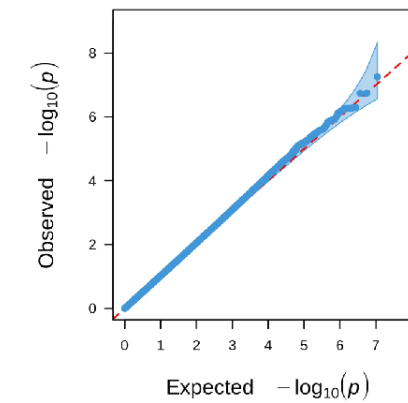

Supplement: Supplementary file 1 [file genes-14-02129-s001.zip › Supplementary files/Figure S2.pdf]
